# Ideal timing to transfer from an acute care hospital to an interdisciplinary inpatient rehabilitation program following a stroke: an exploratory study

**DOI:** 10.1186/1472-6963-6-151

**Published:** 2006-11-23

**Authors:** Dany Gagnon, Sylvie Nadeau, Vincent Tam

**Affiliations:** 1 École de réadaptation, Faculté de médecine, Université de Montréal, Montréal, Québec, Canada; 2 Centre de recherche interdisciplinaire en réadaptation, Institut de réadaptation de Montréal, Montréal, Québec, Canada; 3 Hôpital de réadaptation Lindsay, 6363 chemin Hudson, Montréal, Québec, H3S 1M9, Canada

## Abstract

**Background:**

Timely accessibility to organized inpatient stroke rehabilitation services may become compromised since the demand for rehabilitation services following stroke is rapidly growing with no promise of additional resources. This often leads to prolonged lengths of stays in acute care facilities for individuals surviving a stroke. It is believed that this delay spent in acute care facilities may inhibit the crucial motor recovery process taking place shortly after a stroke. It is important to document the ideal timing to initiate intensive inpatient stroke rehabilitation after the neurological event. Therefore, the objective of this study was to examine the specific influence of short, moderate and long onset-admission intervals (OAI) on rehabilitation outcomes across homogeneous subgroups of patients who were admitted to a standardized interdisciplinary inpatient stroke rehabilitation program.

**Methods:**

A total of 418 patients discharged from the inpatient neurological rehabilitation program at the Montreal Rehabilitation Hospital Network after a first stroke (79% of all cases reviewed) were included in this retrospective study. After conducting a matching procedure across these patients based on the degree of disability, gender, and age, a total of 40 homogeneous triads (n = 120) were formed according to the three OAI subgroups: short (less than 20 days), moderate (between 20 and 40 days) or long (over 40 days; maximum of 70 days) OAI subgroups. The rehabilitation outcomes (admission and discharge Functional Independence Measure scores (FIM), absolute and relative FIM gain scores, rehabilitation length of stay, efficiency scores) were evaluated to test for differences between the three OAI subgroups.

**Results:**

Analysis revealed that the three OAI subgroups were comparable for all rehabilitation outcomes studied. No statistical difference was found for admission (P = 0.305–0.972) and discharge (P = 0.083–0.367) FIM scores, absolute (P = 0.533–0.647) and relative (P = 0.496–0.812) FIM gain scores, rehabilitation length of stay (P = 0.096), and efficiency scores (P = 0.103–0.674).

**Conclusion:**

OAI does not seem to affect significantly inpatient stroke rehabilitation outcomes of patients referred from acute care facilities where rehabilitation services are rapidly initiated after the onset of the stroke and offered throughout their stay. However, other studies considering factors such as the type and intensity of the rehabilitation are required to support those results.

## Background

The demand for rehabilitation services following stroke is rapidly growing since the population at risk for vasculocerebral accident is continuously increasing and the progress in acute clinical management have resulted in higher survival rates over the past few years [1]. Due to limited financial and specialized human resources, the publicly-funded and universal-access health care system is challenged by this increasing demand for inpatient stroke rehabilitation services [2]. Eventually, rapid accessibility to organized inpatient stroke rehabilitation services may become compromised, especially during the initial recovery period, and may lead to a prolonged length of stays in acute care facilities for individuals surviving a stroke [3,4]. It is believed that this potential unnecessary delay spent in acute care facilities, commonly refer to as nonmedical bed-days (not resulting from medically unstable acute or chronic comorbidity), may inhibit the crucial motor recovery process taking place shortly after a stroke [5,6]. Over the past few years, the development of comprehensive and interdisciplinary stroke units in most acute care facilities deserve additional attention since it may influence rehabilitation outcomes [7]. Physical therapy, occupational therapy, and speech and language therapy services are now rapidly initiated after the onset of the stroke and offered throughout the acute care hospital stay [7]. On these specialized interdisciplinary units, therapeutic interventions target specific goals to be achieved according to specific treatment plan developed for each patient [7]. As the stroke survivors become medically stable, therapy intensity usually increases in preparation for the transfer to an inpatient stroke rehabilitation program [7].

The association between the timing of rehabilitation efforts and rehabilitation outcomes has been repeatedly documented [8-12]. Numerous studies have concluded that individuals with stroke who are admitted earlier to inpatient stroke rehabilitation programs usually present the most favourable functional recovery [8-10]. However, this association has been challenged given evidences showing that the time elapsed between the onset of the stroke and the admission to inpatient rehabilitation program is rather a strong indicator (confounder) of the health status of individuals who suffered a stroke [11,12]. Clinically, stroke survivors, who are medically ready for discharge earlier, usually experienced only minor decline in the integrity of their motor, sensory or cognitive functions [11,12]. As a consequence, these patients may be admitted sooner to inpatient rehabilitation and may reach the most favourable rehabilitation outcomes, in terms of degree of disability and discharge destination. Age- and gender-specific variability have also been identified as strong prognostic factors influencing stroke rehabilitation outcomes [13-15].

It is important to document the ideal timing to initiate intensive inpatient stroke rehabilitation after the neurological event. Therefore, the objective of this study was to examine the specific influence of short, moderate and long onset-admission intervals (OAI) on rehabilitation outcomes across homogeneous subgroups of patients who were admitted to a standardized interdisciplinary inpatient stroke rehabilitation program.

## Methods

### Setting

The Montreal Rehabilitation Hospital Network (MRHN) is formed by five publicly-funded rehabilitation hospitals offering standardized interdisciplinary inpatient neurological rehabilitation programs. A total of 749 patients were admitted to the multiple rehabilitation hospitals of the MRHN neurology program offering similar rehabilitation treatments during the study (12-month period) from acute care facilities where inpatient rehabilitation services (physical therapy, occupational therapy, speech and language therapy) are rapidly initiated after admission (usually within 72 hours). The majority of these patients presented with a primary diagnosis of stroke (534 individuals). Admission to these programs is reserved to individuals who experience residual impairments or disabilities following a recent stroke, remain hospitalized in an acute care hospital, and demonstrate a capability to actively participate and tolerate daily intense therapeutic interventions (physical, occupational, speech and language therapies) that are usually initiated within 24 hours from admission. Despite the paucity of beds available in relation to the demand for services, tremendous efforts are made to assure rapid admission to a rehabilitation hospital, usually within 72 hours after the request, to minimize nonmedical bed-days among inpatient rehabilitation candidates waiting in acute care facilities. Rehabilitation hospitals can accommodate to specific needs of each medically stable patient. Readiness for inpatient rehabilitation discharge depends on personal needs of each individual in conformity with their plan of treatment and is periodically reviewed by the interdisciplinary team with input from the patient and its family when indicated.

### Subjects

Following approval of this study by the Research and Ethics Committees, medical records were reviewed retrospectively for the 534 clients who registered in the MRHN neurology program with a primary diagnosis of stroke based on clinical history, neurological evaluation or neuroradiologic studies. Patients were excluded if they presented associated medical conditions influencing the course of rehabilitation (previous stroke, post-cardiac surgery [16], lower extremity amputation or recent hip fracture), had incomplete medical records, were returned to acute care facilities due to medical complications, died during rehabilitation or simply signed out of rehabilitation despite medical advice. The final sample of this retrospective cohort study consisted of 418 clients discharged from the inpatient neurological rehabilitation program after a first stroke (79% of all cases reviewed). Incomplete medical records explain the majority of the exclusions in this study.

### Onset-admission intervals

The onset-admission interval (OAI) was defined as being the number of days elapsed between the onset of the stroke and admission to the interdisciplinary inpatient stroke rehabilitation program. All participants were classified into a short (less than 20 days), moderate (between 20 and 40 days) or long (over 40 days; maximum of 70 days) OAI subgroups. A similar classification has been previously used in a stroke study conducted in Italy [8]. Although a period of 20 days may not appear as a short OAI in certain countries [17], other recent studies have even defined a period of less than 30 days as being a short OAI (early admission) [18,19].

### Functional assessment

The Functional Independence Measure™ (FIM) [20], which is the most widely accepted functional assessment measure in the rehabilitation community [21], was completed by trained rehabilitation professionals to assess the functional capacity at admission to and at discharge from inpatient rehabilitation for all subjects. The conceptual basis of this instrument is to determine the type and amount of human assistance required by a person with impairment and disability to effectively perform basic activities of daily living. The FIM consists of 18 items organized under six categories of function: self-care activities, sphincter control, mobility, locomotion, communication and social integration. Each item is scored on a standardized ordinal scale from one (completely dependent) to seven (fully independent) for a maximum potential total score of 126. This measurement instrument also allows the calculation of motor-FIM subscore derived from 13 items describing physical abilities and cognitive-FIM subscore which highlights communication and social cognition abilities. The motor-FIM and cognitive-FIM subscores range from 13 to 91 and from 5 to 35, respectively. The motor-FIM subscore has been found to provide a discriminative measure when evaluating stroke rehabilitation outcomes [22].

### Degree of disability

All subjects selected were classified into one of 14 Inpatient Rehabilitation Facility-Case Mix Groups (IRF-CMG), specifically developed for individuals with a diagnosis of stroke, based on a Classification and Regression Trees statistical methodology (Table 1) [23]. These 14 groups are primarily structured on the basis of clinical characteristics known on admission to rehabilitation (motor-FIM, cognitive-FIM, age). Theoretically, expected resource needs may be determined and resource use homogeneity may be assured for each specific CMG (homogeneous groups) in all rehabilitation facilities. Frequent applications of CMGs are found in development of specific lengths of stay, payment models, outcome measures and benchmarking initiatives [24]. The tub/shower transfer score was removed from the motor score equation in the IRF-CMG classification system since measured performance level were positively correlated with costs as opposed to most of the other items and that transfer-to-tub question does not measure an absolute level of function in its current wording [25]. In fact, this score could fluctuate depending on architectural configuration of the environment and adaptive equipment available to clients. For this reason, motor dimension of the FIM ranges from 12 to 84 in the IRF-CMG classification structure instead of the usual 13 to 91 points.

### Matching procedure

A block design with a matching procedure was used to control for the covariant effects that degree of disability (IRF-CMGs), gender and age may have on the OAI, and ultimately on the rehabilitation outcomes. Three matching variables were selected: IRF-CMGs (14 levels), gender (male or female) and age (within 3 years). Each subject was identified by a IRF-CMG, gender, and age. Subjects were selected from each OAI subgroup to generate triads. When multiple subjects were identified within the same OAI subgroups, subjects were matched automatically with the first subject who presented similar characteristics (age, gender, CMG) until no more triads could be created. Subjects for whom there was no exact severity of disability (CMG), age, and gender counterparts were excluded. Given the fact that 120 subjects were selected overall, the final data set consisted of 40 homogeneous triads (Table 2) after conducting the matching procedure for IRF-CMG, gender and age (n = 40 for the short, moderate and long OAI subgroups). Participants included in the short, moderate and long OAI subgroups were found to be well representative of the entire stratum from which they were selected as no difference was found for the age, gender and stroke severity (CMG) between the groups (P = 0.226–0.924).

### Rehabilitation outcomes

The following information was collected for analysis:

#### FIM scores

Total, motor and cognitive-FIM scores were measured as previously described.

#### FIM change scores

Calculations for changes in functional status (total-FIM, motor-FIM, and cognitive-FIM) made between admission and discharge were determined using an absolute method of calculation which highlights the absolute difference in raw scores between admission and discharge scores. Similarly, relative FIM changes were also calculated (Discharge FIM-Admission FIM/Admission FIM).

#### Length of stay

The length of stay (LOS) corresponds to the net number of days elapsed between admission and discharge from inpatient stroke rehabilitation. This measure is often referred to as the duration of the inpatient rehabilitation stay and represent a useful rehabilitation cost indicator [26].

#### Efficiency scores

The total, motor, and cognitive-efficiency scores report the absolute change in FIM score divided by the inpatient rehabilitation LOS. This ratio represents the average amount of FIM gain per day during inpatient rehabilitation.

### Data analysis and statistics

Descriptive statistics, expressed as mean and one standard deviation (SD), were calculated for the continuous data (age, OAI, LOS) for the total sample and the three OAI subgroups. Between-group differences for participants with different OAI intervals were calculated using a one-way independent-samples analysis of variance (ANOVA) for the age, OAI, and LOS after verifying the heterogeneity of variances. When the ANOVA was significant, Tukey post-hoc testing was conducted to locate the differences between the short, medium, and long OAI subgroups. Descriptive statistics, expressed as 25^th^, 50^th ^(median) and 75^th ^percentiles, were calculated for the categorical data (total, motor and cognitive-FIM scores at admission and discharge; total, motor and cognitive-FIM absolute and relative gain scores; total, motor and cognitive-efficiency scores) for the total sample and the three OAI. For these rehabilitation outcomes, between-group differences for participants with different OAI intervals were calculated using a Kruskal-Wallis analysis for non-parametric data. A level of significance of 0.05 was selected for all tests. All data were analyzed using the SPSS^® ^11.5 statistical analysis software.

## Results

The final sample included 120 patients equally distributed into three OAI subgroups of each 40 patients: short, moderate, and long (Table 3). As expected, mean OAI was significantly different across each OAI subgroup (*P *< 0.001) whereas no mean age difference was observed between the OAI subgroups (*P *= 0.934). The LOS was not significantly affected by the timing at which inpatient rehabilitation was initiated (*P *= 0.096) although patients with a long OAI had a tendency to have a longer LOS when compared to those with short or moderate OAI.

Results of admission and discharge functional assessments along with the absolute and relative changes measured during inpatient rehabilitation are summarized in Table 4. Comparable admission total (*P *= 0.828), motor (*P *= 0.972), and cognitive-FIM scores (*P *= 0.305) were observed between the OAI subgroups. Likewise, comparable discharge total (*P *= 0.083), motor (*P *= 0.108), and cognitive-FIM (*P *= 0.367) scores were found between the OAI subgroups. Both the absolute and relative total (*P *= 0.647 and 0.812), motor (*P *= 0.533 and 0.725), and cognitive FIM change scores (*P *= 0.610 and 0.496) were comparable between the OAI subgroups, respectively.

Finally, total, motor, and cognitive-efficiency scores generated from total, motor and cognitive absolute FIM gain scores and inpatient rehabilitation LOS measured for all patients and for the short, medium and long OAI subgroups are presented in Table 4. Similar total (*P *= 0.120), motor (*P *= 0.103), and cognitive-efficiency scores (*P *= 0.674) were reached between the OAI subgroups.

## Discussion

Inpatient rehabilitation outcomes (FIM scores, FIM change scores, LOS, FIM efficiency score) were not influenced by the OAI in this study. Surprisingly, the impact of the OAI on stroke rehabilitation outcomes was found to be suppressed when controlling for the degree of severity, gender, and age. These results challenge the consensus that stroke survivors who are rapidly admitted to inpatient rehabilitation potentially reach more favourable outcomes than those admitted later, and the presumption that delayed inpatient stroke rehabilitation admission may prompt adverse effect on rehabilitation outcomes. Overall, these results confirm that if interdisciplinary rehabilitation services are rapidly initiated after the stroke, OAI loses importance independently of the setting (acute hospital vs rehabilitation centre) although a small effect of timing (OAI) might still remain (Efficiency scores: FIM total *P *= 0.120; FIM motor *P *= 0.103). This small effect definitively deserves attention as this can be of clinical importance even if no statistically significant difference was found. This trend corroborates that individuals who are medically stable following a first CVA should rapidly be transferred to a rehabilitation facility offering an intensive stroke rehabilitation program. Under no circumstance should the admission to a rehabilitation facility from an acute care facility be delayed.

Both publicly-funded and universal-access acute care and rehabilitation hospitals may not be as proactive at initiating discharge procedures as privately-funded healthcare institutions since less constraints (e.g.: financial) influence this process. In fact, the average OAI of 30.97 (SD 18.12) days calculated in this study was almost three times more elevated than the average of 11 days recently reported in the United-States of America (USA) following a stroke [17]. This reduced OAI reported in the USA may result, in part, from the presence of numerous constrained for admission to rehabilitation (e.g.: financial) and from the limited number of severe cases (FIM-Total score lower than 40) admitted to rehabilitation facilities despite evidence of slow recovery among these individuals. Highly disabled stroke clients are likely to undergo unfavourable outcome, but unexpected improvement cannot be ruled out [27]. Nonetheless, adherence to specific validated criteria to determine readiness for medical discharge following a stroke may lead to a reduction of the OAI, which may in turn contribute to cost reductions in acute care hospital [5,28,29]. Concerning the inpatient stroke rehabilitation LOS, it is obvious that the publicly-funded and universal-access health care system, as seen in Canada, also permits a more prolonged LOS during inpatient rehabilitation than what is reported in the USA. For example, the average LOS of 50.81 (SD 24.51) days calculated in this study was more than two times the average of 20 (SD 13) days recently reported in the USA following a stroke [17]. Reasons similar to those supporting reduced OAI in the USA may also partially explain the short LOS observed in this country. A reduced LOS may definitively have an effect on the efficiency scores reported in this study. Since limited financial and human resources available currently prevent the expansion of inpatient stroke rehabilitation programs, refinement of their discharge criteria, which would influence the LOS, may simultaneously optimise inpatient stroke rehabilitation efficiency and improve accessibility to these programs. Rapid access to rehabilitation programs for all individuals who sustain a stroke should never be jeopardized from an ethical, biological, clinical or administrative point of view.

Strength of rehabilitation interventions offered in acute care facilities also deserves additional attention to better determine its impact on OAI of clients referred to inpatient rehabilitation programs [30]. This study demonstrates that OAI did not have a significant impact on rehabilitation LOS (*P *= 0.096) among individuals with stroke. Yet, the fact that individuals with a long OAI remained almost 10 days longer than those with short or moderate OAI in rehabilitation may represent a clinically and administrative relevant difference. Moreover, there is no evidence suggesting that rehabilitation is ineffective if begun at a later time. In fact, recent publication even proposes that rehabilitation in chronic stroke clients should not be neglected and represents a promising option [31].

It is clear that inpatient rehabilitation after a stroke is effective for all individuals although variability is observed among OAI groups. All patients equally gained from inpatient stroke rehabilitation indifferently of OAIs although individuals with short OAI had an extra 8 total-FIM units than those with moderate or long OAI (*P *= 0.083). More precisely, the comparable FIM efficiency scores found between the OAI subgroups suggest that similar rates of functional improvement may be expected despite differences in OAI. Alternative services, such as ambulatory hospital-based rehabilitation services or early supported discharge with home rehabilitation, may represent interesting clinical approaches to limit the OAI (nonmedical bed days), especially for individuals with mild disability [32,33]. This definitively needs to be further studied since superior therapeutic efficacy and effectiveness may be reached during these programs when compared to inpatient stroke rehabilitation.

Since it is still extremely difficult to discriminate functional gains emerging from spontaneous recovery from those resulting from therapeutic interventions, caution is advised when analyzing the various levels of therapeutic efficiency measured. Spontaneous recovery might occur at different times for individuals with various degree of disability following a stroke and might overlap with recuperation attributable to therapeutic interventions (rehabilitation and adaptation processes). It is believed that spontaneous recovery usually results from three separate, but interactive, processes which include resolution of diaschisis (loss of function in remote areas anatomically connected to region of lesion), behavioural compensation and neuroplasticity [34,35]. More precisely, the mechanisms underlying the neuroplasticity has been variously attributed to redundancy (parallel distributed pathways), changes in synaptic strength, axonal sprouting with formation of new synapses, assumption of function by contralateral homologous cortex, and substitution of uncrossed pathways [36].

Some evidences suggest a possible association between the severity of the vasculocerebral event and the duration of the spontaneous recovery process [37]. Courses of spontaneous recovery process associated to rapid initiation of rehabilitation efforts might fluctuate among individuals presenting various levels of disability following a stroke, refuting the general belief that early rehabilitation is associated to greater gains for all of them. As highlighted by this study, this general belief may be modulated by the severity of impairment and disability levels following a stroke. Consequently, the timing of intensive functional rehabilitation deserves more attention to ensure best possible cumulative gains resulting from spontaneous recovery and therapeutic interventions. Other elements such as purity, specificity, dose, intensity, duration, and timing of rehabilitation interventions also require added consideration to better understand treatment efficiency in stroke rehabilitation [30].

### Study limitations

Although this study further advocate the efficiency of inpatient stroke rehabilitation programs, one must be prudent in interpreting these results since the retrospective design of this study, combined to the modest sample size, are inherent limitations. Future prospective study would need to document functional status (FIM scores) at consistent times following stroke between the OAI groups to remove this potential confounding effect. Possible ceiling and bottom effects of the FIM measurement instrument selected to assess functional capacity are also well documented [38]. Clinically, admission scores on the FIM could be lower than expected (underscore) among individuals with severe disability and higher than expected among those with mild one representing a possible bias [39]. Moreover, measurements of therapeutic specificity and intensity at the acute care and rehabilitation hospitals should definitively be included in future prospective study given their possible influence on rehabilitation LOS and efficiency [40,41]. The documentation of depression, when present, that may lead to loss of motivation or hopelessness about recovery could also be beneficial since it may pose barriers to accomplishing optimal rehabilitation outcomes [42]. Finally, knowledge of the satisfaction level of all patients would bring additional insights to future study [43].

## Conclusion

After controlling for the degree of disability, gender, and age, this matched block design study showed that OAI may not be a relevant prognostic factor of inpatient stroke rehabilitation outcomes for patients referred from acute care facilities where rehabilitation services are rapidly initiated after a stroke. The impacts of early or delayed initiation of inpatient rehabilitation following a stroke may not favourably or adversely affect rehabilitation outcomes, respectively. However, it certainly remains critical to rapidly initiate rehabilitation services after the onset of a stroke at the acute care hospital, according to specific goals to be achieved for each patient, because any delay may adversely influence rehabilitation outcomes. Perhaps most importantly, these results may have important implications in the continuum of care of individuals with stroke between acute care and rehabilitation hospitals. Finally, the result of the present study not only contrasts from previous literature but also supports the need of future prospective studies focusing on the influence of OAI on inpatient stroke rehabilitation outcomes and their retention, even improvement, over time after discharge.

## Competing interests

The author(s) declare that they have no competing interests.

## Authors' contributions

DG made substantial contributions to the conception and design of this study, to the data acquisition, statistical analysis, interpretation of the results and to the writing of this manuscript. SN participated to the statistical analysis and the interpretation of the results of this study and helped to draft the manuscript. VT was involved in the conception of the study and in drafting and reviewing the manuscript. Following a critical review, all authors read and approved the submitted manuscript.

## Pre-publication history

The pre-publication history for this paper can be accessed here:



## Figures and Tables

**Table 1:** Description of the Inpatient Rehabilitation Facility- Case-Mix Group (IRF-CMG) classification system for individuals with stroke

IRF-CMG	FIM motor score* (12–84)	FIM cognitive score (5–35)	Age (years)
0101 Stroke	69–84	23–35	-
0102 Stroke	59–68	23–35	-
0103 Stroke	59–84	5–22	-
0104 Stroke	53–58	-	-
0105 Stroke	47–52	-	-
0106 Stroke	42–46	-	-
0107 Stroke	39–41	-	-
0108 Stroke	34–38	-	≥ 83
0109 Stroke	34–38	-	≤ 82
0110 Stroke	12–33	-	≥ 89
0111 Stroke	27–33	-	82–88
0112 Stroke	12–26	-	82–88
0113 Stroke	27–33	-	≤ 81
0114 Stroke	12–26	-	≤ 81

* Tub/shower transfer score not included

**- **No restriction related to this variable for this IRF-CMG

**Table 2:** Number of subjects stratified by severity of disability and gender characteristics in each OAI subgroups used for the composition of the age-matched homogeneous triads

Classification	Entire Group		Short OAI		Moderate OAI		Long OAI		Homogeneous triads^a^	
	
	Male	Female	Male	Female	Male	Female	Male	Female	Male	Female
Total	209	209	117	112	67	71	25	26	19	21
										
IRF-CMG										
101	57	52	30	34	24	16	3	2	3	2
102	35	36	27	25	6	9	2	2	1	2
103	12	12	4	3	5	4	3	5	3	2
104	27	20	18	11	6	7	3	2	3	2
105	17	21	13	11	2	8	2	2	0	2
106	10	11	3	4	4	4	3	3	2	2
107	11	11	6	5	3	4	2	2	0	2
108	0	1	0	1	0	0	0	0	0	0
109	8	10	3	4	3	4	2	2	2	2
110	0	0	0	0	0	0	0	0	0	0
111	3	2	3	0	0	2	0	0	0	0
112	3	2	0	1	3	0	0	1	0	0
113	14	21	5	8	7	8	2	5	2	5
114	12	10	5	5	4	5	3	0	3	0

^a^Matched homogeneous triads formed for each CMG-, age- and gender-criteria (n = 40)

**Table 3:** Mean (1SD) of age, onset-admission interval (OAI) and length of stay (LOS) of participants

Outcomes	Entire Group (n = 120)	Short OAI (n = 40)	Moderate OAI (n = 40)	Long OAI (n = 40)	*P*
	Mean (SD)	Mean (SD)	Mean (SD)	Mean (SD)	
Age (years)	71.31 (8.01)	71.47 (8.18)	70.9 (8.50)	71.53 (7.51)	0.934
OAI (days)	30.97 (18.12)	13.18 (3.75)	26.93 (6.17)	52.80 (10.93)	< 0.001*
LOS (days)	50.81(24.51)	47.90 (24.64)	46.90 (21.08)	57.63 (26.63)	0.096

* Statistically significant

**Table 4:** Admission and discharge FIM scores, FIM change scores, and efficiency scores measured during inpatient stroke rehabilitation

Outcomes	Entire Group (n = 120)			Short OAI (n = 40)			Moderate OAI (n = 40)			Long OAI (n = 40)			*P*
	
	Percentiles			Percentiles			Percentiles			Percentiles			
	
	25^th^	50^th ^ (median)	75th	25^th^	50^th ^ (median)	75th	25^th^	50^th ^ (median)	75th	25^th^	50^th ^ (median)	75th	
FIM-admission													
Total	61.50	78.00	94.75	61.75	79.00	99.00	57.75	77.00	94.00	57.00	78.00	92.50	0.828
Motor	36.00	50.00	70.75	36.00	49.50	73.25	35.25	50.00	68.25	34.75	50.00	71.50	0.972
Cognitive	20.00	27.00	30.00	21.00	27.50	31.75	19.25	27.00	30.75	18.00	25.50	29.00	0.305
FIM-discharge													
Total	97.00	108.00	117.00	98.25	113.00	119.00	95.25	105.00	115.75	92.50	105.00	111.75	0.083
Motor	69.25	79.00	86.00	72.50	84.50	87.75	66.50	78.50	85.00	69.25	77.50	83.75	0.108
Cognitive	24.25	29.00	32.75	27.00	31.00	33.00	23.00	28.50	33.75	25.00	29.00	31.00	0.367
Change-absolute													
Total	14.00	23.00	37.00	16.25	24.00	39.00	14.00	22.50	34.75	13.25	22.50	33.50	0.647
Motor	11.25	21.50	33.50	12.25	23.50	37.75	11.00	19.50	31.75	10.00	19.50	30.75	0.533
Cognitive	1.00	3.00	5.00	1.00	2.00	4.50	1.00	3.00	5.00	0.00	4.00	5.75	0.610
Change-relative													
Total	16.00	30.20	56.28	18.35	34.10	59.53	15.02	29.20	52.80	14.93	28.60	60.85	0.812
Motor	16.40	43.75	87.40	16.40	51.50	92.63	18.97	35.75	90.98	15.03	41.65	75.60	0.725
Cognitive	3.00	10.00	25.00	3.00	6.90	15.40	3.13	13.10	23.10	0.00	13.10	29.35	0.496
Efficiency													
Total	0.35	0.53	0.77	0.38	0.62	0.83	0.35	0.53	0.71	0.29	0.42	0.66	0.120
Motor	0.27	0.47	0.67	0.35	0.55	0.72	0.26	0.49	0.67	0.20	0.42	0.58	0.103
Cognitive	0.01	0.05	0.11	0.02	0.04	0.10	0.02	0.08	0.13	0.00	0.06	0.11	0.674
